# Supplementary material for: Deletion of wfs1 Impairs Oligodendrocyte Precursor Cells Dorsal Distribution and Myelination Through the wfs1-hmgcs1 Axis in Zebrafish
Source: Biology (Basel). 2026 Aug 16;15(16):1402. doi: 10.3390/biology15161402 (PMC13510151; doi:10.3390/biology15161402)
Supplement: Supplementary file 1 [file biology-15-01402-s001.zip › File S1-Transcriptome sequencing data and differentially expressed genes (DEGs) in wfs1-- zebrafish at 5 dpf/1.GO/wfs1KOvsWT/UP/wfs1KOvsWT.up_GObar_mf.pdf]

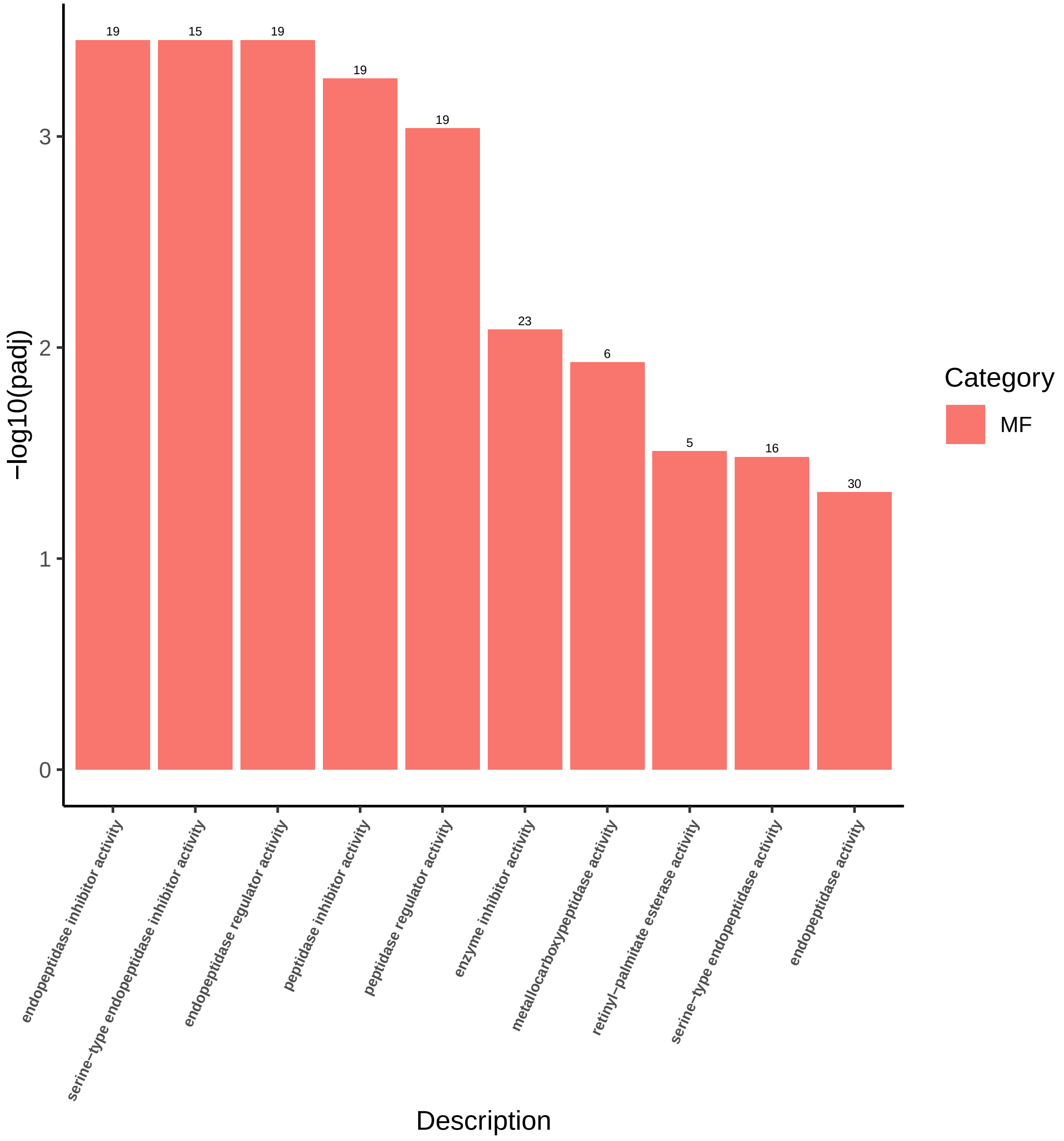
19
15
19
19
19
23
6
5
16
30
−log10(padj)
3
2
1
0
endopeptidase inhibitor activity
serine−type endopeptidase inhibitor activity
endopeptidase regulator activity
peptidase inhibitor activity
peptidase regulator activity
enzyme inhibitor activity
metallocarboxypeptidase activity
retinyl−palmitate esterase activity
serine−type endopeptidase activity
endopeptidase activity
Description
Category
MF
